# Sb-Doped Cerium Molybdate: An Emerging Material as Dielectric and Photocatalyst for the Removal of Diclofenac Potassium from Aqueous Media

**DOI:** 10.3390/molecules28247979

**Published:** 2023-12-06

**Authors:** Ayesha Javaid, Muhammad Imran, Farah Kanwal, Shoomaila Latif, Syed Farooq Adil, Mohammed Rafi Shaik, Mujeeb Khan

**Affiliations:** 1 Centre for Inorganic Chemistry, School of Chemistry, University of the Punjab, Lahore 54000, Pakistan; 2 Centre for Physical Chemistry, School of Chemistry, University of the Punjab, Lahore 54000, Pakistan; 3 School of Physical Sciences, University of the Punjab, Lahore 54000, Pakistan; 4 Department of Chemistry, College of Science, King Saud University, P.O. Box 2455, Riyadh 11451, Saudi Arabia

**Keywords:** cerium molybdate, doping, antimony, dielectric, AC conductivity, photocatalysis, diclofenac

## Abstract

This work reports the influence of antimony substitution in a cerium molybdate lattice for improved dielectric and photocatalytic properties. For this purpose, a series of Ce_2−x_Sb_x_(MoO_4_)_3_ (x = 0.00, 0.01, 0.03, 0.05, 0.07, and 0.09) were synthesized through a co-precipitation route. The as-synthesized materials were characterized for their optical properties, functional groups, chemical oxidation states, structural phases, surface properties, and dielectric characteristics using UV–Vis spectroscopy (UV–Vis), Fourier transform infrared (FTIR) and Raman spectroscopies, X-ray photoelectron spectroscopy (XPS), X-ray diffraction (XRD), Brunauer–Emmett–Teller (BET) analysis, and impedance spectroscopy, respectively. UV–Vis study showed a prominent red shift of absorption maxima and a continuous decrease in band gap (3.35 eV to 2.79 eV) by increasing the dopant concentration. The presence of Ce–O and Mo–O–Mo bonds, detected via FTIR and Raman spectroscopies, are confirmed, indicating the successful synthesis of the desired material. The monoclinic phase was dominant in all materials, and the crystallite size was decreased from 40.29 nm to 29.09 nm by increasing the Sb content. A significant increase in the dielectric constant (ε′ = 2.856 × 10^8^, 20 Hz) and a decrease in the loss tan (tan*δ* = 1.647, 20 Hz) were exhibited as functions of the increasing Sb concentration. Furthermore, the photocatalytic efficiency of pristine cerium molybdate was also increased by 1.24 times against diclofenac potassium by incorporating Sb (x = 0.09) in the cerium molybdate. The photocatalytic efficiency of 85.8% was achieved within 180 min of UV light exposure at optimized conditions. The photocatalytic reaction followed pseudo-first-order kinetics with an apparent rate constant of 0.0105 min^−1^, and the photocatalyst was recyclable with good photocatalytic activity even after five successive runs. Overall, the as-synthesized Sb-doped cerium molybdate material has proven to be a promising candidate for charge storage devices and a sustainable photocatalyst for wastewater treatment.

## 1. Introduction

Human civilization strives hard to flourish, but it is the rule of Mother Nature that whenever the balance between technology and policy is disturbed, destruction often follows. This scenario has led to several issues like energy depletion and environmental deterioration, which have become critical challenges of this century worldwide. The increased demand for energy supply requires novel energy sources and efficient energy storage systems. According to an estimate, the energy storage market is an emerging sector with a net worth of USD 210 billion in 2021 and is expected to reach around USD 400 billion by 2030. Moreover, global energy storage demand in 2022 is estimated at 222.79 GW and is expected to reach 512.41 GW by 2030 [1]. The other prominent issue of high concern is environmental pollution, of which water pollution is ranked at the top. Due to the poor water quality, about one-third of the world’s population is facing water scarcity and cannot access clean drinking water. As per the World Health Organization (WHO) report, millions of deaths are reported annually due to diarrhea caused by drinking unclean water and inappropriate sanitation [2]. These statistical data highlight the dire need to address the global issues mentioned above. Efficient energy storage demands a material with superb dielectric properties for supercapacitors, charge storage batteries, and high-frequency devices. In contrast, water remediation requires techniques that can efficiently and cost-effectively remove harmful pollutants. Photocatalysis is one such effective method that brings about the complete mineralization of toxic contaminants into simple, less harmful compounds. However, it requires suitable materials as photocatalysts have appropriate optical band gaps and a slow recombination of charge carriers [3]. 

Material science has brought incredible breakthroughs in exploring new materials with diverse functionalities, in which the same material can be utilized for multiple purposes. Recently, nanoscale metal oxides have been reported to be highly effective in catalysis, biological activities, sensors, optoelectronics, solar cells, and energy storage devices. The remarkable physicochemical characteristics, high chemical stability, good surface functionalities, large specific surface area, continuous absorption, and intensive/narrow emission spectra are some of the top-notch qualities of nanoscale materials, which make them distinct from their bulk counterparts [4]. Among various metal oxides, rare earth molybdates, especially cerium molybdate, have attracted the attention of researchers owing to their sizeable electronic conductivity, stable crystal structure, good optical absorption, and appreciable physical/chemical properties. These properties are attributable to the unique electronic transitions of incompletely occupied 4f and unoccupied 5d orbitals in trivalent rare earth cations, improving catalytic activity and charge storage potential [5]. For instance, Muthuvel and coworkers reported hydrothermally synthesized cerium molybdate for the photocatalytic removal of Fuschin dye, and it removed 92% of dye within 90 min of solar light irradiation [6]. Similarly, Dargahi et al. reported the surfactant-assisted microemulsion synthesis of cerium molybdate nanoparticles for the photocatalytic removal of crystal violet dye with a degradation efficiency of 89%, achieved after five h of visible light illumination [5]. Tian et al. reported a good charge storage ability of cerium molybdenum oxide-based ceramics with a high dielectric constant of 10.69 and small current leakage with a slight loss tan value of 1.88 × 10^−4^ [7]. The literature emphasizes the need to explore this material further to increase its dielectric constant for charge storage properties and to enhance its photocatalytic activity toward the degradation of different pollutants. However, this multifunctional attribute with improved characteristics demands modulations in its fundamental structure, which can be effectively achieved via doping. A comprehensive literature review shows that the substitution of some of the atoms of the host material with other metal cations of comparable radii and similar oxidation states results in structural imperfections that could enhance the electronic and catalytic response of the material [8]. Among different metals, the primary group metals with ns^2^ lone pairs of electrons (Sn, Sb, and Bi) have gained enormous attention from researchers, especially antimony (Sb). It is an electrically active dopant known to significantly increase the concentration of charge carriers, enhance oxygen vacancies, and improve optoelectronic properties [9]. These assumptions, supported by the recent reports on the use of antimony doping to enhance the characteristics of different molybdates, motivated us to use Sb as a dopant for cerium molybdates [10,11]. 

**Figure 1:**
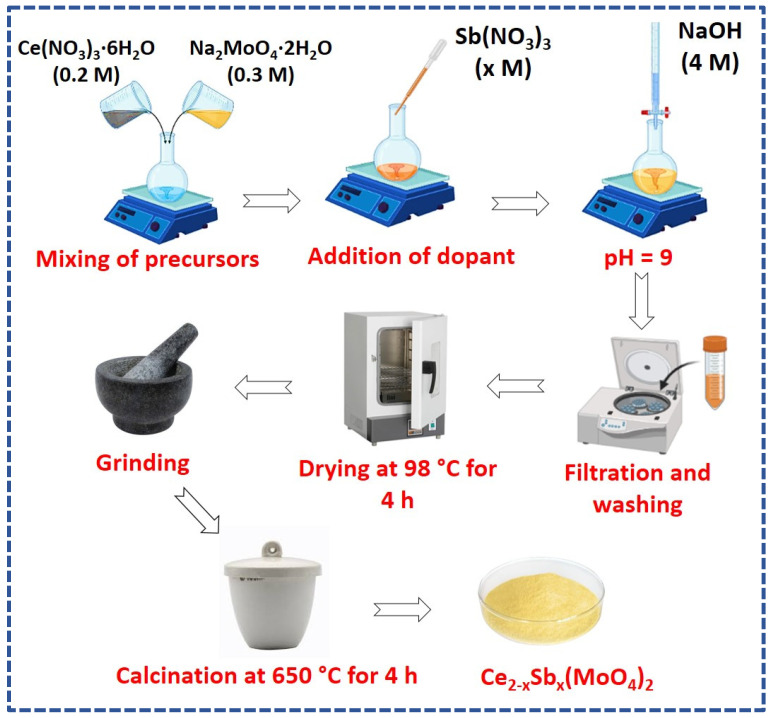
Schematic representation of Ce_2−x_Sb_x_(MoO_4_)_2_ (0.00 ≤ x ≤ 0.09) synthesis via co-precipitation method.

Thus, the present work is designed to synthesize antimony-doped cerium molybdate via a co-precipitation method (Figure 1). Co-precipitation is a quick and homogenous synthetic route that produces small particles in less time, utilizes only a tiny external energy input, provides reasonable control over doping level, and requires simple equipment. The variable amounts of antimony were substituted in cerium molybdate lattice, and different characterization techniques were employed to investigate the effect of the dopant on the structural/optical/textural properties of cerium molybdate. The study of dielectric constant, loss tan, AC conductivity, and electric modulus analysis provided insight into fabricated materials’ charge storage potential. Moreover, the photocatalytic potential of these materials was also explored against diclofenac potassium. The optimized reaction conditions, reaction kinetics, and plausible photodegradation mechanism are also included in this manuscript. 

## 2. Results and Discussion

### 2.1. UV–Visible Study

**Figure 2:**
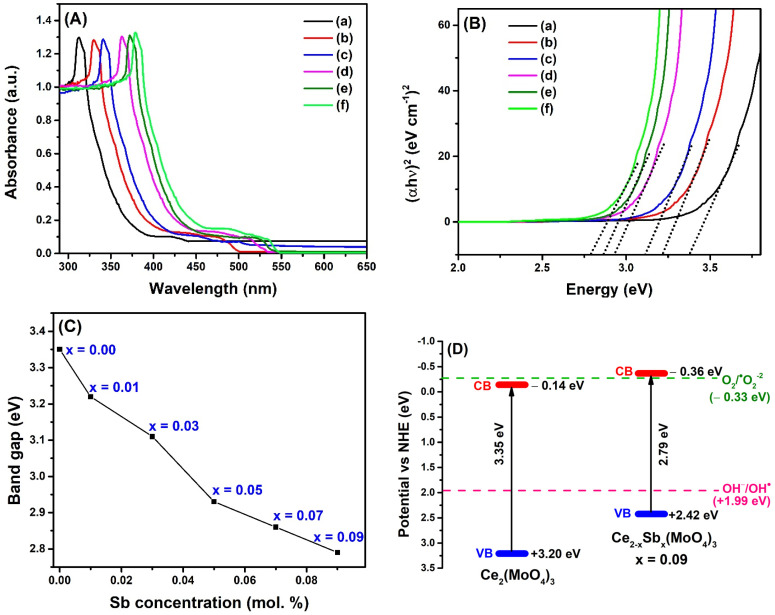
(**A**) UV–Vis absorption spectra; (**B**) optical band gaps of (a) Ce_2_(MoO_4_)_3_, (b) Ce_2–x_Sb_x_(MoO_4_)_3_ (x = 0.01), (c) Ce_2−x_Sb_x_(MoO_4_)_3_ (x = 0.03), (d) Ce_2−x_Sb_x_(MoO_4_)_3_ (x = 0.05), (e) Ce_2−x_Sb_x_(MoO_4_)_3_ (x = 0.07), and (f) Ce_2−x_Sb_x_(MoO_4_)_3_ (x = 0.09); (**C**) variation of band gap with variable dopant concentration; and (**D**) band edge potentials of Ce_2_(MoO_4_)_3_ and Ce_2−x_Sb_x_(MoO_4_)_3_ (x = 0.09).

The optical properties of synthesized materials were studied through UV–visible spectroscopy. The variations of absorption maximum were recorded by changing wavelengths in the range of 250–700 nm. All the materials showed good absorption in the UV region, owing to the outer orbital electronic transitions of cerium and molybdenum (Figure 2A). The pristine cerium molybdate showed λ_max_ = 312 nm, whereas absorption edges of Ce_2–x_Sb_x_(MoO_4_)_3_ (x = 0.01), Ce_2−x_Sb_x_(MoO_4_)_3_ (x = 0.03), Ce_2−x_Sb_x_(MoO_4_)_3_ (x = 0.05), Ce_2−x_Sb_x_(MoO_4_)_3_ (x = 0.07), and Ce_2−x_Sb_x_(MoO_4_)_3_ (x = 0.09) were centered at 330 nm, 341 nm, 362 nm, 371 nm, and 378 nm, respectively. A bathochromic shift was observed in the absorption edges as antimony was added to the host material. This red shifting of the absorption band by increasing the antimony concentration indicated the distortion of the host lattice, which could tune the optical band gaps [12]. The UV–Vis absorption data were used to calculate the optical band gap energies of synthesized materials using the Tauc relation (Equation (1)), according to which, the optical band gap is related to the absorbance of material and the energy of incident photons:

(αhν) = A(hν − E_g_)^n^, (1)

where α is the absorption coefficient, A represents the proportionality constant, h is Planck’s constant, ν is the frequency of the absorbed photon, E_g_ denotes the optical band gap, and n is a variable that depends on the kind of electronic transition (*n* = 1/2, 2, 3/2, or 3 for allowed direct, allowed indirect, forbidden direct, or forbidden indirect, respectively). The molybdate family is known to have natural allowed transitions, i.e., *n* = 1/2. The graph was plotted between photon energy (hν) on the *x*-axis and (αhν)^2^ on the *y*-axis (Figure 2B). The linear part of the curve was extrapolated to the *x*-axis until (αhν)^2^ = 0, and at this point, the value of the *x*-axis provided optical band gaps of the synthesized materials. The plot shows that when hν < E_g,_ the plot is parallel to the *x*-axis. In contrast, when hν > E_g_, the curve rises linearly with an increase in the photon energy (hν), probably caused by the diffusion of electrons from the valence band to the conduction band of the material due to energy absorption. The calculated band gaps were 3.35 eV, 3.22 eV, 3.11 eV, 2.93 eV, 2.86 eV, and 2.79 eV for Ce_2_(MoO_4_)_3_, Ce_2–x_Sb_x_(MoO_4_)_3_ (x = 0.01), Ce_2−x_Sb_x_(MoO_4_)_3_ (x = 0.03), Ce_2−x_Sb_x_(MoO_4_)_3_ (x = 0.05), Ce_2−x_Sb_x_(MoO_4_)_3_ (x = 0.07), and Ce_2−x_Sb_x_(MoO_4_)_3_ (x = 0.09), respectively, indicating that band gaps were reduced by increasing dopant concentration (Figure 2C). The observed decrease in band gap upon Sb doping is consistent with the literature, where holmium/ytterbium doping is reported to decrease the band gap of cerium molybdate [13]. The optical bandgap data were used to further determine the positions of band edge potentials of Ce_2_(MoO_4_)_3_ and Ce_2−x_Sb_x_(MoO_4_)_3_ (x = 0.09). The respective potentials of the valence band (VB) and conduction band (CB) concerning the standard hydrogen potential were calculated using Equations (2) and (3):

E_VB_ = X − E_0_ + 0.5E_g_, (2)

E_CB_ = E_VB_ − E_g_, (3)

where E_VB_ and E_CB_ signify the potential edges of VB and CB, respectively. X is the Mulliken electronegativity of the photocatalysts and E_0_ corresponds to the energy of free electrons on the hydrogen scale (∼4.5 eV vs. NHE). The obtained band structures are illustrated in Figure 2D. The calculated E_VB_ potentials of Ce_2_(MoO_4_)_3_ and Ce_2−x_Sb_x_(MoO_4_)_3_ (x = 0.09) were 3.20 eV and 2.42 eV, and the resultant E_CB_ potentials were −0.14 eV and −0.36 eV, respectively. It can be noticed that Sb doping not only narrowed the optical band gap, but also favorably shifted the E_CB_ and E_VB_ toward standard redox potentials of OH• and •$O_{2}^{-}$, indicative of the more remarkable photocatalytic ability of the Ce_2−x_Sb_x_(MoO_4_)_3_ (x = 0.09) [14]. 

### 2.2. FTIR Analysis

**Figure 3:**
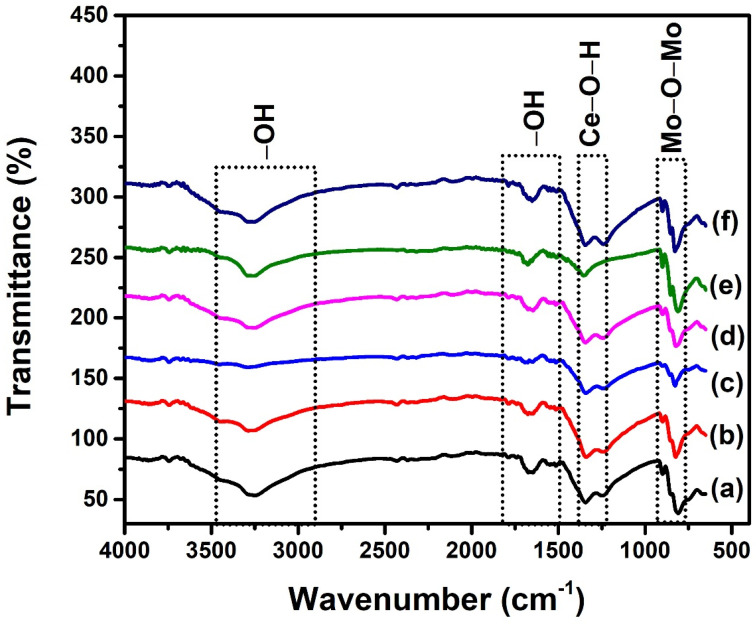
FTIR spectra of (a) Ce_2_(MoO_4_)_3_, (b) Ce_2−x_Sb_x_(MoO_4_)_3_ (x = 0.01), (c) Ce_2−x_Sb_x_(MoO_4_)_3_ (x = 0.03), (d) Ce_2−x_Sb_x_(MoO_4_)_3_ (x = 0.05), (e) Ce_2−x_Sb_x_(MoO_4_)_3_ (x = 0.07), and (f) Ce_2−x_Sb_x_(MoO_4_)_3_ (x = 0.09).

The FTIR spectral studies were used to estimate the bond formations in the antimony-doped cerium molybdate by comparing spectra of cerium molybdate and doped cerium molybdate in the range of 4000 to 625 cm^−1^ (Figure 3). The two small bands that appeared at 807 cm^−1^ (ν_1_) and 845 cm^−1^ (ν_2_) were attributed to the stretching modes of Mo–O–Mo bonds, whereas a sharp peak (ν_3_) observed at 910 cm^−1^ was due to the vibrations of tetrahedral MoO_4_ ions of cerium molybdate [15]. A bathochromic shift was observed for doped materials for all bands (ν_1_, ν_2_, and ν_3_) as the dopant concentration increased. The observed trend could be explained by the shortening of metal–oxygen bond lengths caused due to the incorporation of dopant [16]. Since the cerium ions possessing an ionic radius of 1.14 Å were replaced by small-sized Sb ions (0.76 Å), a greater strength of the bond was expected, which led to the shortened bond length, which is in agreement with the reported findings [17]. The band (ν_4_) observed in the range of 1350–1220 cm^−1^ specified the bending modes of M–O–H (M = Ce) [18]. The broad spectral bands observed in the range of 3400–3100 cm^−1^ and 1600–1650 cm^−1^ were due to the stretching and bending vibration of hydroxyl groups of adsorbed water molecules [19]. The observed IR bands are inconsistent with the reported IR spectra of cerium molybdate [18,20]. No separate peaks were observed for antimony oxide, which demonstrated that the host lattice was substituted by all of the antimony and was found to agree with the previously reported work on antimony-doped nanostructures [21].

### 2.3. Raman Spectroscopy

**Figure 4:**
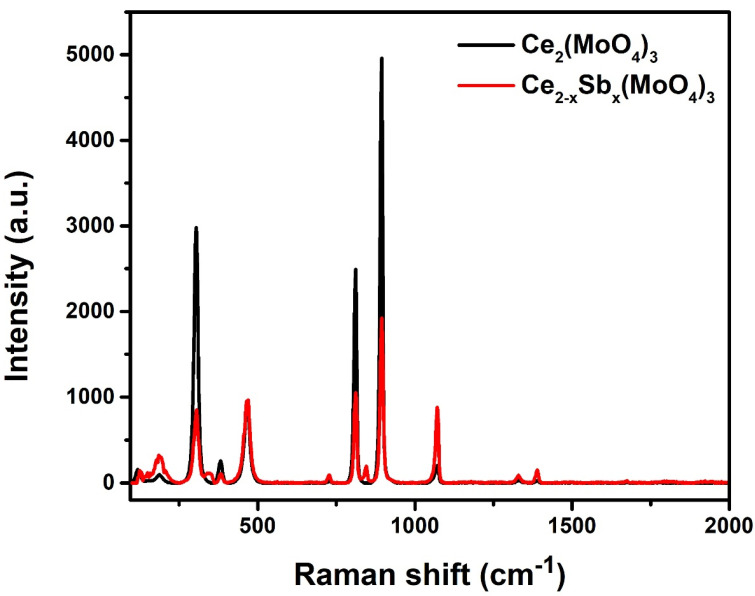
Raman spectra of Ce_2_(MoO_4_)_3_ and Ce_2−x_Sb_x_(MoO_4_)_3_ (x = 0.09).

The Raman spectra of synthesized Ce_2_(MoO_4_)_3_ and Ce_2−x_Sb_x_(MoO_4_)_3_ (x = 0.09) were recorded in the range of 100 cm^−1^–2000 cm^−1^ as shown in Figure 4. The Raman shift at 900 cm^−1^ and 815 cm^−1^ were attributed to bridging vibrations of O–Mo–O and Mo–O, respectively. The band at 467 cm^−1^ showed symmetric vibrations of Ce–O bonds [22], whereas the Raman band at 304 cm^−1^ specified the symmetric bending modes of Mo–O [20]. The peak intensity of cerium and molybdenum vibrations decreased after Sb substitution, implying the presence of imperfect lattice sites after adding the dopant. This can also be attributed to the change in the efficient mass of oscillating atoms due to modifications in crystallite size and lattice distortions in O–Mo–O and O–Ce–O bonds [23]. 

### 2.4. XPS Study

**Figure 5:**
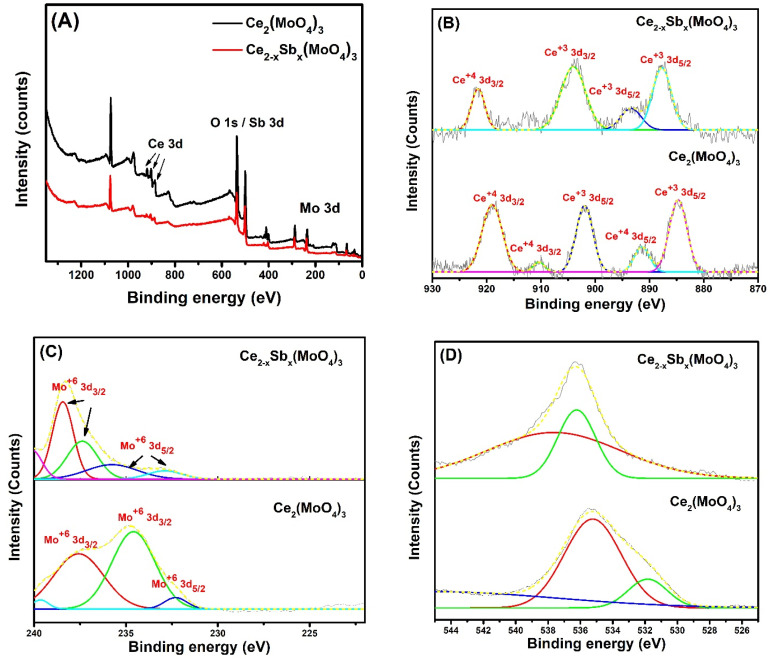

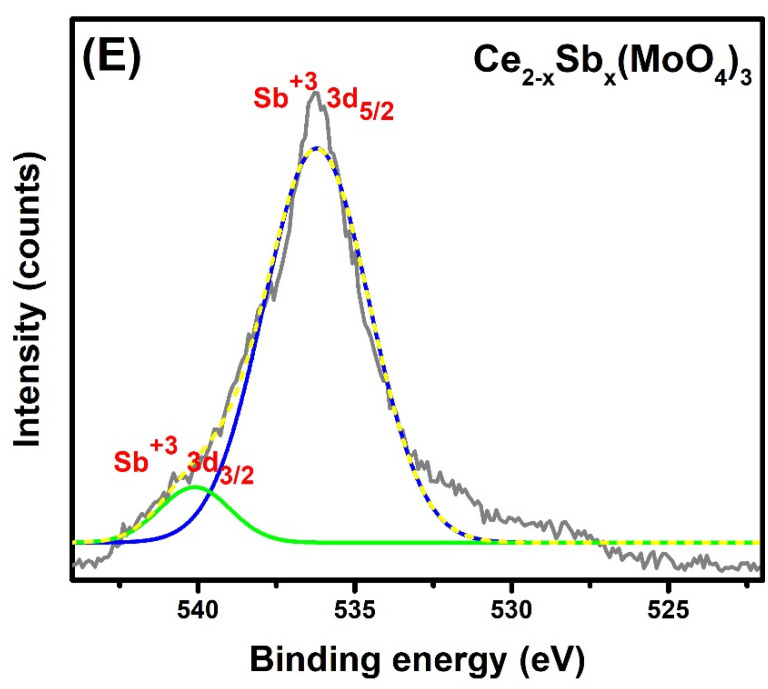
(**A**) XPS survey; (**B**) XPS spectra of Ce 3d, (**C**) XPS spectra of Mo 3d, and (**D**) XPS spectra of O 1s of Ce_2_(MoO_4_)_3_ and Ce_2−x_Sb_x_(MoO_4_)_3_ (x = 0.09); (**E**) XPS spectrum of Sb 3d of Ce_2−x_Sb_x_(MoO_4_)_3_ (x = 0.09) (dotted line—fitted curves; colored lines—deconvoluted peaks).

The chemical composition of as-fabricated materials was studied via X-ray photoelectron spectroscopy (XPS). The survey spectra of Ce_2_(MoO_4_)_3_ and Ce_2−x_Sb_x_(MoO_4_)_3_ (x = 0.09) demonstrated the respective signals of cerium, molybdenum, oxygen, and antimony, as shown in Figure 5A, and confirmed the formation of desired compositions. The Sb 3d could not be observed clearly in the full XPS spectrum due to its small dosage and overlapping with O 1s peaks. The high-resolution XPS spectra of Ce 3d showed two deconvoluted peaks at 885.12 eV and 901.93 eV ascribed to the 3d_5/2_ and 3d_3/2_ splitting of Ce with +3 oxidation state (Figure 5B) [24]. The other three peaks which appeared at a binding energy of 891.31 eV (3d_5/2_), 910.35 eV (3d_3/2_), and 918.64 eV (3d_3/2_) showed the presence of Ce in the +4 state due to the oxidation of Ce^+3^. The occurrence of some cerium in the +4 state agrees with the previously reported work [25]. For Sb-doped cerium molybdate (Ce_2−x_Sb_x_(MoO_4_)_3_ (x = 0.09)), three out of four deconvoluted peaks were observed for Ce^+3^ at binding energies of 887.81 eV (3d_5/2_), 893.29 eV (3d_5/2_), and 904.15 (3d_3/2_), whereas only one peak at 921.09 eV depicted the presence of Ce in +4 oxidation state with spin–orbit splitting of 3d _3/2_ [26]. It was evident from Figure 5B that signals of Ce^+4^ have been reduced via Sb doping, and only one peak appeared for Ce^+4^, having a smaller peak area than the peak obtained for undoped cerium molybdate and showing that antimony doping has restrained the oxidation of Ce^+3^ into Ce^+4^. The core-level XPS spectrum of Mo, deconvulated into three prominent peaks, appeared at 232.2 eV, 234.5 eV, and 237.5 eV (Figure 5C). The first peak was ascribed to the spin–orbital splitting of Mo 3d_5/2_, whereas the other two peaks were due to the Mo 3d_3/2_ and suggested the presence of molybdenum in the +6 oxidation state [25]. There was a minor shift in these peaks upon antimony doping, with some additional peaks appearing at 235.01 eV (Mo 3d_5/2_) and 238.21 eV (Mo 3d_3/2_) [27]. Figure 5D manifests the high-resolution XPS spectra of O 1s with a wide range of peaks appearing in the range of 530–535 eV (Figure 4D). The low binding energy peaks belong to the oxygen vacancies in the cerium molybdate lattice, whereas high energy peaks originated due to the lattice oxygen present in the form of Ce–O and Mo–O species in the fabricated materials [27,28]. The XPS core-level spectrum of Sb 3d showed a characteristic broad peak for antimony in the range of 530–540 eV, as depicted in Figure 5E. This broad peak was deconvulated into two peaks; one, of a more significant intensity peak, appeared at a binding energy of 536.26 eV due to Sb 3d_5/2_ splitting [29], and the other peak of smaller intensity appeared at 540.02 eV ascribed to Sb 3d_3/2_ [30]. According to previous reports, the appeared peaks indicated a +3 oxidation state of Sb in the synthesized Ce_2−x_Sb_x_(MoO_4_)_3_ (x = 0.09) sample and confirmed the apparent incorporation of Sb^+3^ into the cerium molybdate matrix [30].

### 2.5. XRD Analysis

**Figure 6:**
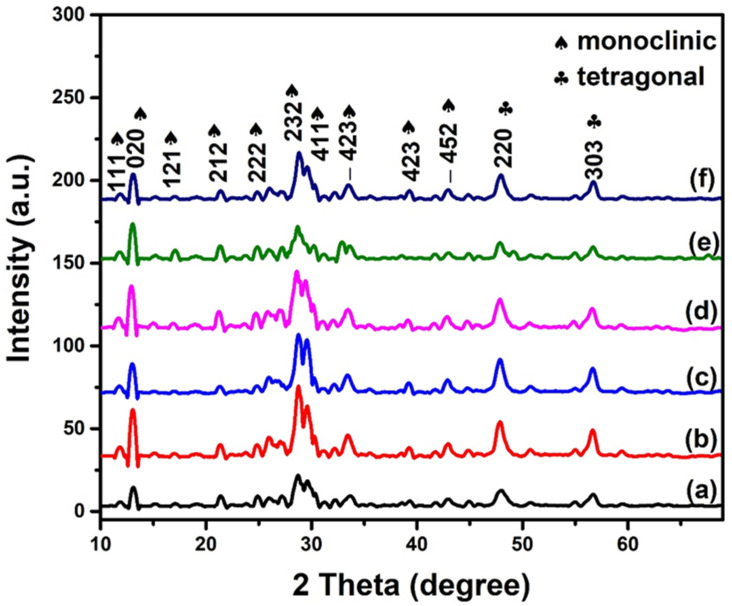
XRD spectra of (a) Ce_2_(MoO_4_)_3_, (b) Ce_2−x_Sb_x_(MoO_4_)_3_ (x = 0.01), (c) Ce_2−x_Sb_x_(MoO_4_)_3_ (x = 0.03), (d) Ce_2−x_Sb_x_(MoO_4_)_3_ (x = 0.05), (e) Ce_2−x_Sb_x_(MoO_4_)_3_ (x = 0.07), and (f) Ce_2−x_Sb_x_(MoO_4_)_3_ (x = 0.09).

The XRD patterns of synthesized materials were recorded at room temperature to identify the phases present in the samples, as depicted in Figure 6. It shows well-defined sharp peaks for all synthesized materials, suggesting well-crystalized and ordered structures over a long range. The prominent peaks which appeared at 12.06°, 13.20°, 16.74°, 21.62°, 22.49°, 23.09°, 24.56°, 26.02°, 26.80°, 27.31°, 28.40°, 28.82°, 29.64°, 30.18°, 31.20°, 32.26°, 33.13°, 33.72°, 34.69°, 39.19°, 41.70°, 42.94°, and 44.19° were indexed to the lattice planes of (111), (020), (121), (212), (131), (–213), (222), (123), (–313), (140), (–331), (232), (411), (133), (–224), (402), (214), (–423), (–234), (423), (016), (352), and (–452), respectively. These peaks were well matched with the standard diffraction pattern of JCPDS Card No. 01–081–1155, indicating the monoclinic phase of cerium molybdate with the P21/c space group. The monoclinic phase of synthesized cerium molybdate was found to be in agreement with the studies reported by Brazdil et al., Jakab–Costenoble et al., and Sena et al. [31,32,33]. However, two low-intensity peaks that appeared at 48.01° and 56.75° due to the tetragonal phase were indexed to the (220) and (303) planes, respectively [20]. It has also been observed that the peak positions were slightly shifted toward a lower 2θ value in doped samples, indicating the incorporation of Sb in the host cerium molybdate structure. No other peaks were observed for individual phases of cerium oxide, molybdenum oxide, or antimony oxide, revealing the purity of synthesized material. The percentage crystallinity was also evaluated to estimate the degree of structural order in the synthesized materials. Table 1 shows that Sb-doped materials have a higher crystallinity than pristine cerium molybdate. The high crystallinity may result in an efficient charge transfer and reduce the availability of recombination centers for charge carriers, which could result in enhanced photocatalytic activity [34].

The average crystallite sizes (D) of the synthesized materials were calculated using the Debye–Scherrer equation (Equation (4)):

D = kλ/βcosθ,(4)

where k is a Scherrer constant equal to 0.94, λ represents the wavelength of incident X-rays (λ = 0.154 nm), β denotes the full-width half maximum of high-intensity peaks, and θ is the peak position at a certain Bragg’s angle. The calculated crystallite sizes of Ce_2_(MoO_4_)_3_, Ce_2−x_Sb_x_(MoO_4_)_3_ (x = 0.01), Ce_2−x_Sb_x_(MoO_4_)_3_ (x = 0.03), Ce_2−x_Sb_x_(MoO_4_)_3_ (x = 0.05), Ce_2−x_Sb_x_(MoO_4_)_3_ (x = 0.07), and Ce_2−x_Sb_x_(MoO_4_)_3_ (x = 0.09) were 40.29 nm, 36.86 nm, 34.98 nm, 32.63 nm, 31.27 nm, and 29.09 nm, respectively. These results showed a decrease in average crystallite size upon Sb doping as dopant atoms exert a retarding force against crystallite growth. Moreover, the ionic radii of Sb^3+^ (0.76 Å) are smaller than the ionic radii of Ce^3+^ (1.14 Å), which was another contributing factor toward the decreased crystallite size [35]. Other researchers also reported similar findings with different dopants [13,35]. Further, the calculated values of crystallite sizes were used to evaluate the dislocation density (δ) of the samples that measure the number of dislocations in a crystalline material per unit volume of the crystal [36]. It provides information about the defects present in the material and was calculated using Equation (5):

δ = 1/D^2^ (5)

Table 1 shows that an increased dopant concentration increased dislocation density, indicating that the doping introduced distortions and defects in the crystal structure [37]. Doping also causes pressure to build up in crystal lattices due to the foreign atom, which can be evaluated in terms of microstrain. It is a well-known fact that doping is associated with the production of strain in the host lattice. Therefore, the microstrains in the materials were computed using Equation (6) [38]:

ε = βcosθ/4(6)

It was found that Sb doping increased the strain in the sample, which was caused by the variation in the ionic radii of the dopant and host atom [39].

### 2.6. BET Analysis

**Figure 7:**
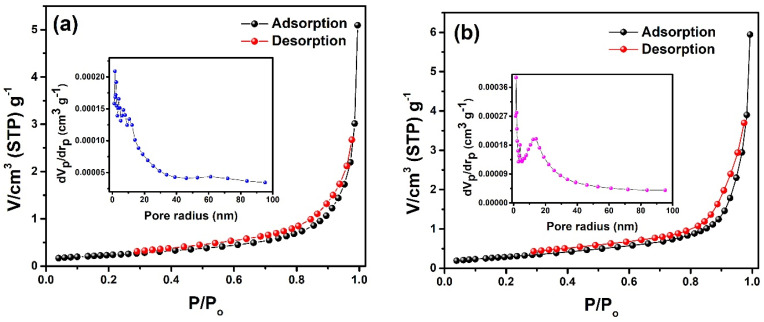

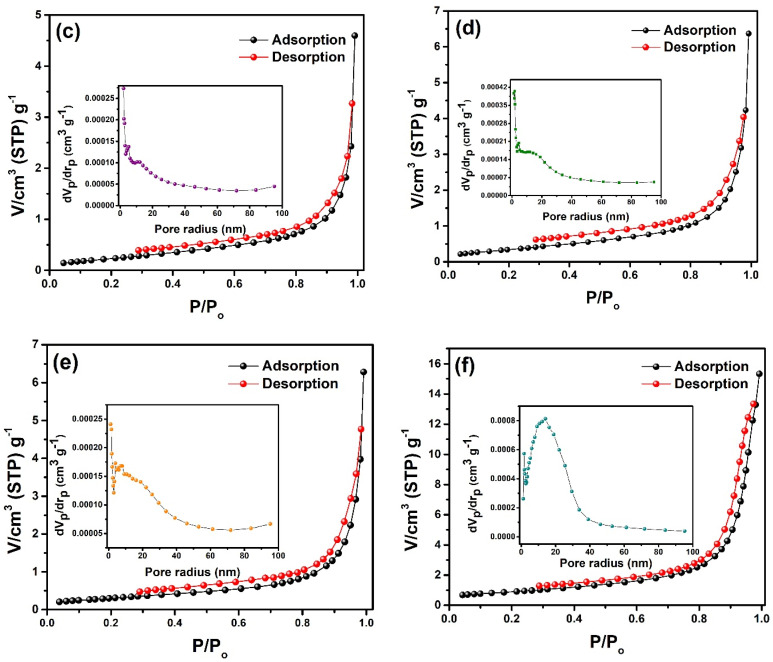
N_2_ adsorption–desorption isotherms and pore volume distribution (inset plots) of (**a**) Ce_2_(MoO_4_)_3_, (**b**) Ce_2−x_Sb_x_(MoO_4_)_3_ (x = 0.01), (**c**) Ce_2−x_Sb_x_(MoO_4_)_3_ (x = 0.03), (**d**) Ce_2−x_Sb_x_(MoO_4_)_3_ (x = 0.05), (**e**) Ce_2−x_Sb_x_(MoO_4_)_3_ (x = 0.07), and (**f**) Ce_2−x_Sb_x_(MoO_4_)_3_ (x = 0.09).

The surface properties of the fabricated materials were studied through BET analysis. All the synthesized materials showed similar adsorption profiles with a constant increase in the nitrogen uptake by increasing the relative pressure, which depicted the porous nature of synthesized materials (Figure 7). The obtained isotherms fitted well with type-III isotherms as per the IUPAC classification, and the materials were classified to be conditionally macroporous, as suggested by the appearance of a hysteresis loop. Moreover, the amount of nitrogen gas desorbed was different from the amount adsorbed, giving rise to an H3 type of hysteresis loop reflecting the slit-shaped pores [40]. It was also found that the amount of nitrogen adsorbed increased from 5.094 cm^3^ g^−1^ to 15.33 cm^3^ g^−1^ by increasing dopant concentration from x = 0.00 to x = 0.09, showing that the material has become somewhat porous due to the addition of the dopant. Literature reports have shown that the porous material can facilitate short diffusion pathways for the movement of ions, which gives rise to a large number of active sites, improving the material’s electrochemical and catalytic properties [41]. Further, the Barrett–Joyner–Halenda (BJH) method was used to investigate the pore size distribution of the samples (Figure 7 (inset)). It also confirmed the mesoporous nature of the samples with the average radius of the pores between 10 and 40 nm [42]. Moreover, it was noticed that pore volume significantly increased from 2.1 × 10^−4^ cm^3^ g^−1^ (Ce_2_(MoO_4_)_3_) to 8.0 × 10^−4^ cm^3^ g^−1^ (Ce_2−x_Sb_x_(MoO_4_)_3_ (x = 0.09)), which was due to antimony substitution, that improved the availability of reactive sites present inside the pores. These findings are in agreement with the work of Lv’s group, who reported an increase in the pore volume of their nanocomposites upon Sb doping [43].

### 2.7. SEM Analysis

**Figure 8:**
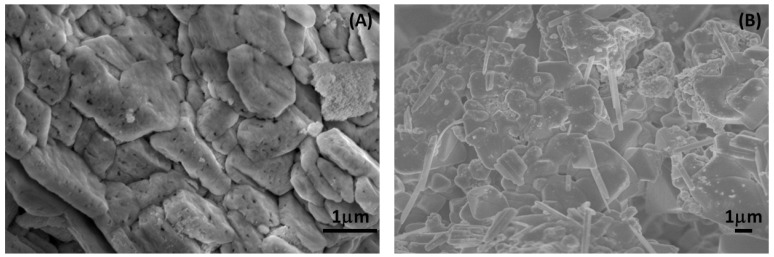
SEM images of (**A**) Ce_2_(MoO_4_)_3_ and (**B**) Ce_2−x_Sb_x_(MoO_4_)_3_ (x = 0.09).

The SEM analysis of the samples was carried out, and the comparative surface morphology was studied. In the sample Ce_2_(MoO_4_)_3_, i.e., the undoped, the surface appeared rugged with undefined morphology; however, it possesses a well-defined grain boundary, along with pores distributed all over the surface. Moreover, there was a difference in the surface morphology observed in the Ce_2−x_Sb_x_(MoO_4_)_3_ (x = 0.09), i.e., the Sb-doped nanocomposite. The surface was rugged and covered with rod-like structures, unlike the undoped compound. In addition, there were no pores found on the surface (Figure 8).

### 2.8. Dielectric Studies

The impedance spectroscopy provides insight into the dielectric properties of the materials. These properties are related to the structural features of the materials, which influence their charge transport and charge storage ability in response to the applied field. Dielectric properties of our materials were investigated in the frequency range of 20 Hz–20 MHz at room temperature. 

#### 2.8.1. Dielectric Constant

**Figure 9:**
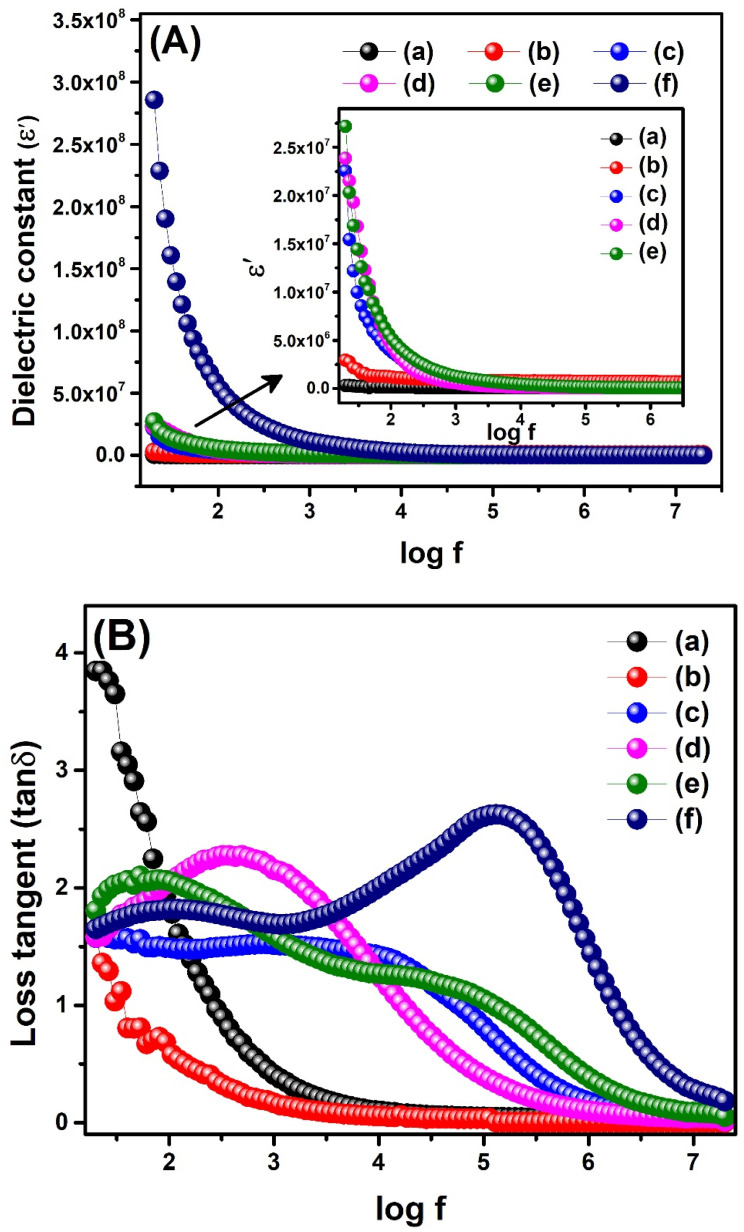

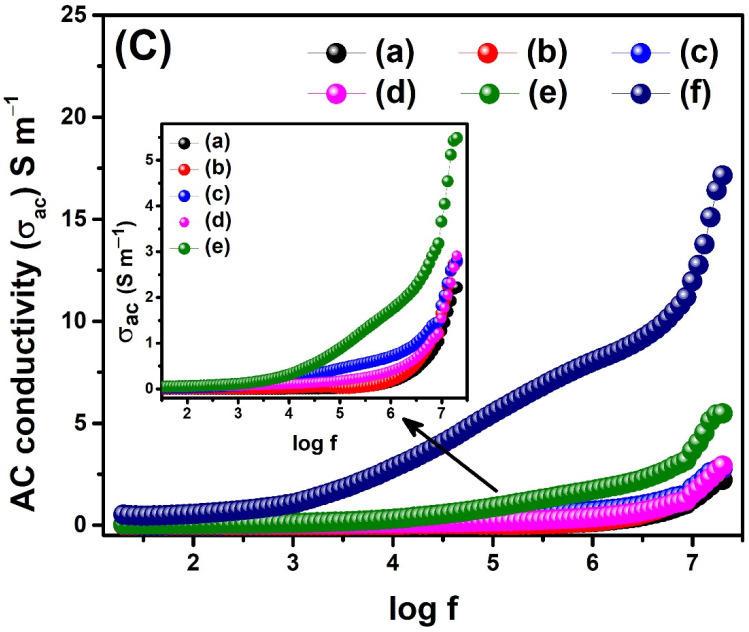
(**A**) Dielectric constant, (**B**) loss tan, and (**C**) AC conductivity of (a) Ce_2_(MoO_4_)_3_, (b) Ce_2−x_Sb_x_(MoO_4_)_3_ (x = 0.01), (c) Ce_2−x_Sb_x_(MoO_4_)_3_ (x = 0.03), (d) Ce_2−x_Sb_x_(MoO_4_)_3_ (x = 0.05), (e) Ce_2−x_Sb_x_(MoO_4_)_3_ (x = 0.07), and (f) Ce_2−x_Sb_x_(MoO_4_)_3_ (x = 0.09).

A material’s dielectric constant (ε′) shows its potential to store electrical energy. The higher the value of the dielectric constant of a material, the greater its potential to store charges, and it can be calculated using the following relation (Equation (7)):

ε′ = C_p_d/ε_o_A, (7)

where ε′ is the dielectric constant, C_p_ represents parallel capacitance, d indicates the thickness of the pallet, ε_o_ is a constant known as permittivity of free space (ε_o_ = 8.84 × 10^−12^), and A signifies the area of the pallet [44]. The variation of ε’ was studied as a function of frequency (20 Hz–20 MHz) and relative dopant content Ce_2−x_Sb_x_(MoO_4_)_3_ (0.00 ≤ x ≤ 0.09) at room temperature (Figure 9A) that displayed the frequency dependence behavior of dielectric constants of synthesized materials. It was evident from the figure that the dielectric constant was appreciably high in the low-frequency region. It sharply decreased upon the increase in frequency and finally became constant. The high values of dielectric constants in low-frequency regimes were attributed to the well-known Maxwell–Wagnet model of interfacial polarization. This model assumes that dielectric materials which consist of conducting grains are separated by relatively less conducting grain boundaries. When the AC field is applied, the permanent and induced dipoles can migrate within conducting grains and orientate themselves correctly due to low frequency. These properly aligned dipoles start accumulating at grain boundaries, which causes sizeable interfacial polarization and, thus, a high dielectric constant [45]. On the other hand, at high frequency, the interfacial polarization starts diminishing as dipoles cannot trail themselves with respect to the enhanced applied AC field; this is known as the polarization relaxation phenomenon. This phenomenon results in low dielectric constant values at high frequencies [46]. The graphical illustration of the influence of various dopant concentrations on the dielectric constant values (Figure 9A) displayed that an increase in dielectric constant was observed by increasing dopant concentration (Table 2). It is well-known that increased voids, dislocations, and defects are responsible for the increased value of the dielectric constant. As evident from XRD, the increase in dopant concentration led to the decreased crystallite size, and an increase in surface defects at grain boundaries was observed. These grain boundaries act as physical barriers to block the flow of charges [47]. The recent work of Benzebeiri et al. also reported an increase in dielectric constant upon Sb doping in multi-ferroic ceramics [48]. 

#### 2.8.2. Dielectric Loss

Loss tangent (tanδ) represents energy dissipation in the form of heat from the dielectric system. Ideally, zero dielectric loss or a relatively small value of dielectric loss is representative of an excellent dielectric material. It occurs when polarization lags behind the applied AC field due to the resistive grain boundaries [49]. The loss tan values of our materials were obtained at room temperature in the frequency range of 20 Hz–20 MHz using the following Equation (8):

tanδ = 1/2πfCR(8)

where f is the applied frequency, C shows parallel capacitance, and R represents resistance [44]. Figure 9B shows that the loss tan is maximum at low frequency and decreases for all samples with increasing frequency. This behavior was supported by the fact that at low frequencies, energy is needed to overcome the internal friction of grain boundaries and to align the dipoles in the direction of the applied field. This results in energy dissipation through dielectric losses from the materials. Conversely, the contribution from resistive grain boundaries decreases at high frequency, and less interfacial polarization leads to small current leakage [45]. The influence of dopant content on the loss tan (Figure 9B) showed that dielectric loss decreased by substituting dopant atoms in the cerium molybdate lattice. The observed loss tan values at 20 Hz were 3.844, 1.583, 1.576, 1.577, 1.807, 1.647 for Ce_2_(MoO_4_)_3_, Ce_2−x_Sb_x_(MoO_4_)_3_ (x = 0.01), Ce_2−x_Sb_x_(MoO_4_)_3_ (x = 0.03), Ce_2−x_Sb_x_(MoO_4_)_3_ (x = 0.05), Ce_2−x_Sb_x_(MoO_4_)_3_ (x = 0.07), and Ce_2−x_Sb_x_(MoO_4_)_3_ (x = 0.09), respectively. The decreased loss tan of doped nanocomposites compared to the pristine cerium molybdate was due to the structural inhomogeneity and defects in the former [45]. However, a slight increase in loss tan of Ce_2−x_Sb_x_(MoO_4_)_3_ (x = 0.07) and Ce_2−x_Sb_x_(MoO_4_)_3_ (x = 0.09) compared to other doped compositions was attributed to their more porous nature than the rest of the doped samples, which provided conducting pathways for current leakage. Another queer behavior was observed in the plot of Ce_2−x_Sb_x_(MoO_4_)_3_ (x = 0.03 to 0.09), which was the appearance of a prominent relaxation peak. This relaxation peak was shifted to a higher frequency region when the dopant concentration was increased, which indicated more potential for a highly doped composition (x = 0.09) to retain charges with small current leakage [50].

#### 2.8.3. AC Conductivity

The total conductivity of a material is the combination of frequency-independent DC conductivity, which occurs due to band conduction, and frequency-dependent AC conductivity due to the hopping of mobile charge carriers between metal ions. The AC conductivity of the synthesized material was studied as a function of frequency and calculated using Equation (9):

σ_ac_ = ω ε′ε_o_ tanδ (9)

where ω, ε′, ε_o_, and tanδ represent angular frequency, dielectric constant, vacuum permittivity, and loss tan, respectively [44]. From the plot of log f vs. AC displayed in Figure 9C, it was noticed that initially, conductivity increases slowly, but only at higher frequencies; there was a sharp rise in conductivity for all synthesized materials. The small value of AC conductivity at low frequency was attributed to the lower availability of mobile charge carriers to hop from one localized state to another caused by the interfacial polarization in this region [51]. At high frequencies, the increased AC conductivity can be attributed to the hopping mechanism, which was strengthened by applying an electric field of higher frequency. The average displacement of charge carriers (mobility of charge carriers) increases at high frequencies, thus increasing electrical conductivity [52]. Moreover, AC conductivity was also appreciably increased by increasing dopant concentration in the composite, which is in concurrence with the previous reports [53,54]. For instance, our pristine cerium molybdate showed an AC conductivity of 2.270 S m^−1^, whereas it increased up to 17.141 S m^−1^ for Ce_2−x_Sb_x_(MoO_4_)_3_ (x = 0.09). The improved AC conductivity upon Sb doping is due to the increased dislocations in the doped samples. These dislocations and defects provide additional sites for electron hopping, thus increasing conductivity [54].

**Table 2:** Dielectric constant (ε′), loss tangent (tanδ), and AC conductivity (σ_ac_) of synthesized compositions of Ce_2−x_Sb_x_(MoO_4_)_3_.

Compositions Ce_2−x_Sb_x_(MoO_4_)_3_	Frequency 20 Hz			Frequency 20 MHz		
	ε′	tanδ	σ_ac_ (S m^−1^)	ε’	tanδ	σ_ac_ (S m^−1^)
x = 0.00	3.263 × 10^5^	3.844	0.00139	7.970 × 10^4^	0.0182	2.270
x = 0.01	2.929 × 10^6^	1.583	0.00516	7.506 × 10^5^	0.0015	2.772
x = 0.03	2.254 × 10^7^	1.576	0.0395	7.779 × 10^4^	0.0114	2.810
x = 0.05	2.385 × 10^7^	1.577	0.0418	7.376 × 10^4^	0.0126	2.914
x = 0.07	2.717 × 10^7^	1.807	0.05459	8.195 × 10^4^	0.0508	5.483
x = 0.09	2.856 × 10^8^	1.647	0.5232	8.441 × 10^4^	0.1826	17.141

A comparison of the dielectric properties of our synthesized materials with previously reported data showed that our Ce_2−x_Sb_x_(MoO_4_)_3_ (x = 0.09) possessed a higher dielectric constant and a slight loss tan value than previously reported ones (Table 3). However, most of the related previously reported materials were studied at much higher frequencies of GHz, making it difficult to compare. Nevertheless, our exceptionally high ε′ and small tanδ in the studied frequency range reflect their potential use in charge storage devices and supercapacitors. 

#### 2.8.4. Electric Modulus Analysis

The relaxation process and transport of electrical charges within a dielectric material can be studied via electric modulus analysis. The real part of the electric modulus (M′) and imaginary part of the electric modulus (M″) were calculated using Equation (10) and Equation (11), respectively:

M′ = ε′/(ε′^2^ + ε″^2^) (10)

M″ = ε″/(ε′^2^ + ε″^2^) (11)

**Figure 10:**
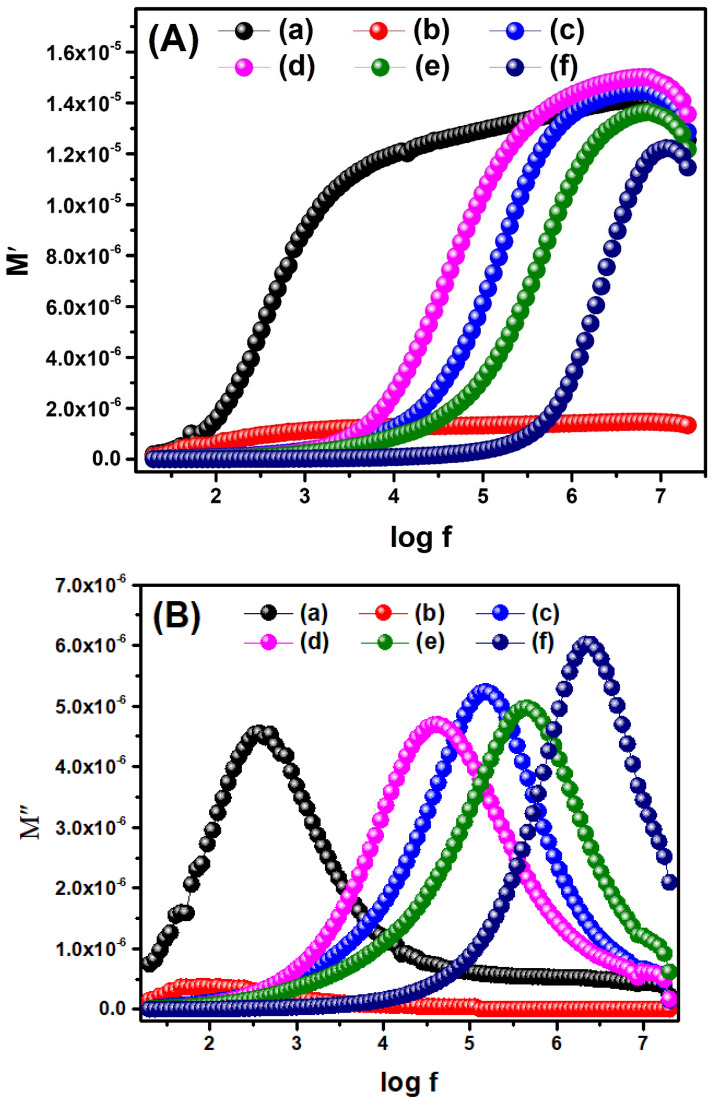

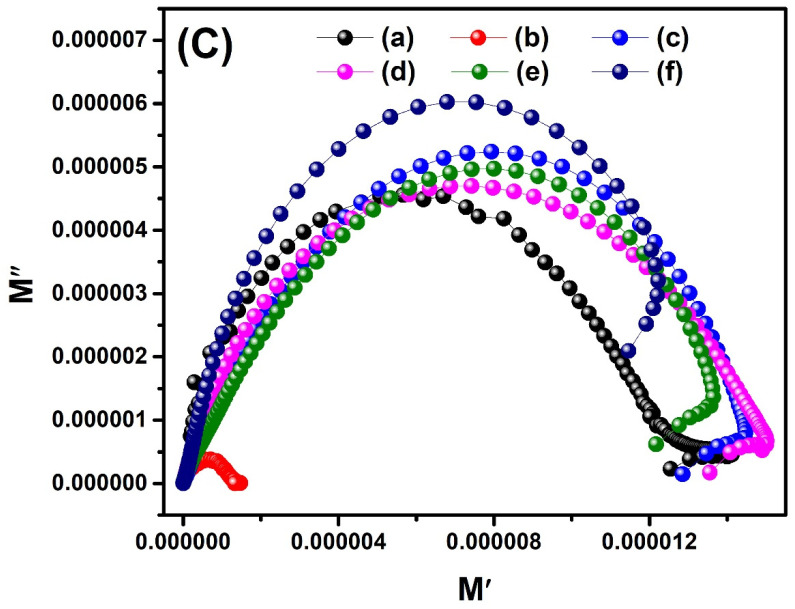
(**A**) Real part of electric modulus; (**B**) imaginary part of electric modulus; and (**C**) Cole–Cole plot of (a) Ce_2_(MoO_4_)_3_, (b) Ce_2−x_Sb_x_(MoO_4_)_3_ (x = 0.01), (c) Ce_2−x_Sb_x_(MoO_4_)_3_ (x = 0.03), (d) Ce_2−x_Sb_x_(MoO_4_)_3_ (x = 0.05), (e) Ce_2−x_Sb_x_(MoO_4_)_3_ (x = 0.07), and (f) Ce_2−x_Sb_x_(MoO_4_)_3_ (x = 0.09).

The values of the obtained real part of the modulus were plotted against log f, as shown in Figure 10A. All samples showed an increase in M′ with an increase in frequency due to the hopping of electrons; thus, the conduction mechanism operates at a high frequency. It was consistent with our findings of the real part of permittivity, where high dielectric constant values were observed at low frequency and vice versa. However, Ce_2_(MoO_4_)_3_ and Ce_2−x_Sb_x_(MoO_4_)_3_ (x = 0.01) indicated a rise in M′ at relatively lower frequencies compared to other samples, showing that conduction started in them even at low frequencies. The rest of the samples showed common constant values of M′ at low frequencies due to ionic polarization. In contrast, at higher frequencies, an asymmetric maximum was observed, which indicates the phenomenon of space charge polarization at this frequency [60]. Furthermore, shifting asymptotic maxima toward a higher frequency by changing dopant concentration shows that the relaxation process has been extended over a long frequency range [61]. 

The frequency and composition-dependent plot of M″ illustrated in Figure 10B displayed only one peak, confirming the phenomenon of single polarization of grain boundaries in all samples [49]. Furthermore, it can be observed that the values of M″ were initially insignificant and then increased with increasing frequency, thereby demonstrating the relaxation phenomenon at higher frequency. Further increase in frequency decreased the M″, revealing a non-Debye type of relaxation in the as-prepared materials. Figure 10B also displays that the relaxation peak was shifted toward a higher frequency by increasing dopant concentration. It indicated a more pronounced Maxwell–Wagner–Sillars polarization mechanism in the doped materials than the undoped one [62]. 

#### 2.8.5. Cole–Cole Plot

The Cole–Cole plot between M′ and M″ helps to explain the role of resistivity of grains and grain boundaries (Figure 10C). Each sample showed a single arc in the form of a perfect semicircle, indicating a single polarization phenomenon; otherwise, deviation from the semicircle could occur due to the involvement of multiple polarization processes. The left side of the semicircle (low-frequency region) is the function of grain resistance. In contrast, the middle part of the semicircle (intermediate-frequency region) is attributed to the resistance due to grain boundaries. The combined effect of grain and grain boundary resistances emerges on the right side of the semicircle (high-frequency region) [63]. The dominance of grain boundary resistance is evident from the appearance of the semicircles for all the as-prepared materials. However, the intensity of the semicircle peak was slightly increased at higher dopant concentrations, revealing the increased grain boundary resistance for these materials [60]. 

### 2.9. Photocatalytic Activity

**Figure 11:**
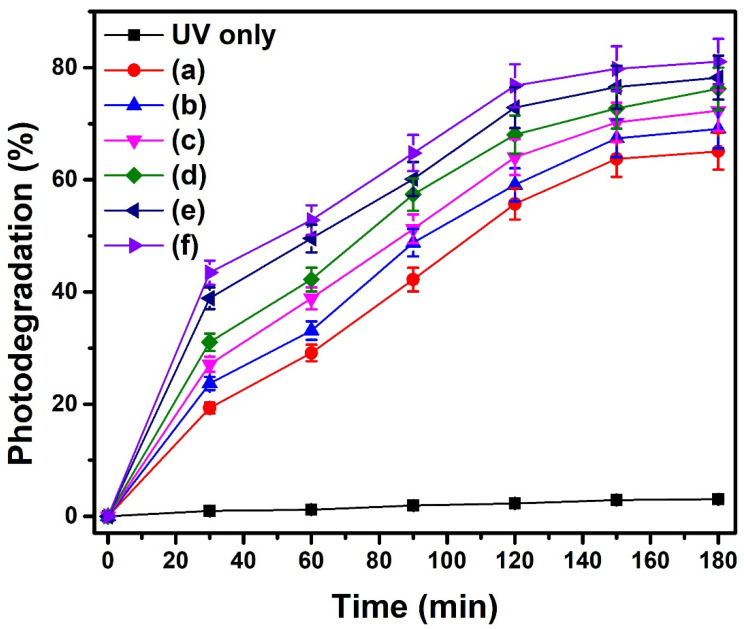
Photocatalytic activity of (a) Ce_2_(MoO_4_)_3_, (b) Ce_2−x_Sb_x_(MoO_4_)_3_ (x = 0.01), (c) Ce_2−x_Sb_x_(MoO_4_)_3_ (x = 0.03), (d) Ce_2−x_Sb_x_(MoO_4_)_3_ (x = 0.05), (e) Ce_2−x_Sb_x_(MoO_4_)_3_ (x = 0.07), and (f) Ce_2−x_Sb_x_(MoO_4_)_3_ (x = 0.09) against diclofenac potassium. (Diclofenac) = 5 mg/L; catalyst dosage = 20 mg and pH = 7.

The photocatalytic degradation activity of the synthesized materials was studied against diclofenac potassium, an anti-inflammatory drug and a pharmaceutical pollutant. The preliminary control experiments were carried out to investigate the individual effects of the catalyst and UV light irradiation separately. It was found that there was no change in drug concentration when the catalyst was used without irradiation, indicating that it was not activated without light. Similarly, irradiation with UV light alone was also insufficient to bring about the degradation of the drug, meaning that photolysis has no role in drug degradation. However, an appreciable decrease in diclofenac concentration was observed when the drug solution was exposed to UV radiation in the presence of the catalyst. The degradation of the drug was recorded in terms of percent degradation and plotted against time, as shown in Figure 11. It was observed that percent degradation increased by increasing the duration of light irradiation and then became nearly constant after a specific time due to the completion of the degradation process. The initial abrupt increase in photodegradation efficiency was attributed to the fact that at the start of the photocatalytic reaction, numerous catalytic sites were available for the catalytic process. With time, the catalytic sites become occupied, and photodegradation becomes slow and finally turns out to be constant. From the graphical illustration, it is observed that the degradation efficiencies were 65.1%, 69.1%, 72.3%, 76.2%, 78.2%, and 80.7% for Ce_2_(MoO_4_)_3_, Ce_2−x_Sb_x_(MoO_4_)_3_ (x = 0.01), Ce_2−x_Sb_x_(MoO_4_)_3_ (x = 0.03), Ce_2−x_Sb_x_(MoO_4_)_3_ (x = 0.05), Ce_2−x_Sb_x_(MoO_4_)_3_ (x = 0.07), and Ce_2−x_Sb_x_(MoO_4_)_3_ (x = 0.09), respectively. It was revealed that photocatalytic activity was enhanced by increasing dopant concentration, which influences the optical, structural, morphological, and textural properties, hence improving the photocatalytic activity [64]. In the present work, the increase in dopant concentration leads to the reduced band gap and crystallite size of pristine cerium molybdate, which resulted in a good catalytic performance of Sb-doped cerium molybdate, especially Ce_2−x_Sb_x_(MoO_4_)_3_ (x = 0.09). To date, no work has been reported regarding the photocatalysis of diclofenac potassium using Sb-doped materials. However, some of the related catalysts used for the photocatalytic degradation of diclofenac potassium are collected and presented in Table 4 to compare the photocatalytic efficiency of the as-prepared material with previously reported ones. 

### 2.10. Optimization

#### 2.10.1. Effect of pH

**Figure 12:**
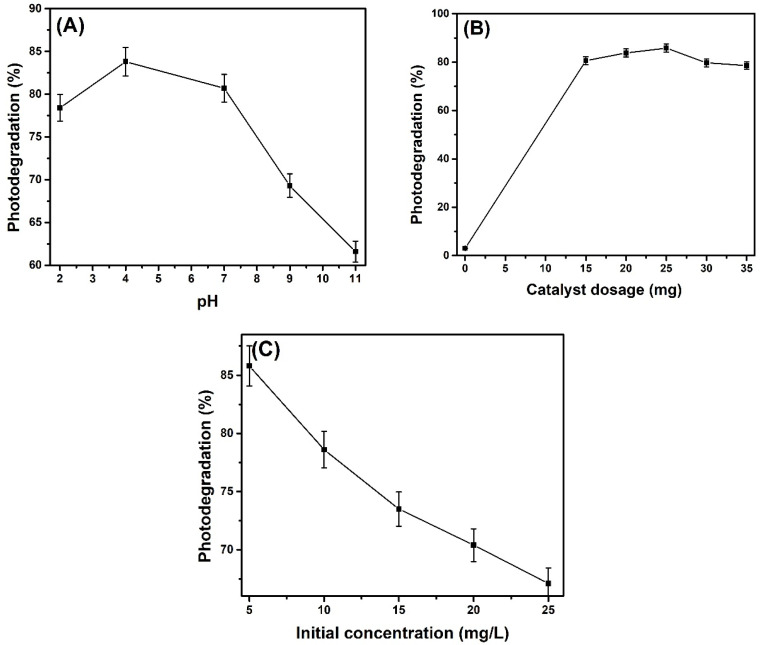
Photocatalytic optimization of (**A**) pH, (**B**) catalyst dosage, and (**C**) initial concentration of diclofenac potassium using Ce_2−x_Sb_x_(MoO_4_)_3_ (x = 0.09) as photocatalyst.

The pH of the photocatalytic reaction is one of the crucial factors as it governs the surface charge properties of a catalyst, which, in turn, affects its photocatalytic efficiency. In our study, the effect of pH was studied by keeping the solution pH in acidic (pH = 2, 4), neutral (pH = 7), and basic (pH = 9, 11) ranges (Figure 12A). From the results obtained, at the highly acidic conditions, i.e., pH = 2, the degradation efficiency was 78.4% and increased to 83.5% at pH = 4. As the pH further increased, the catalytic degradation of the drug started declining, resulting in a photodegradation of 80.7%, 69.4%, and 61.6% at pH = 7, pH = 9, and pH = 11, respectively, which indicated that the acidic conditions caused maximum drug degradation, whereas basic conditions diminished the degradation efficiency of the nanocomposites. Studies found in the literature report that a proton-rich environment under acidic conditions causes excess positive charge on the catalyst’s surface, which develops electrostatic attraction between the anionic drug and the positively charged catalyst’s surface [70]. Nevertheless, this explanation suggests that the degradation efficiency should be more significant at pH = 2 than at pH = 4, but we observed the opposite trend. This could be because the pK_a_ value of diclofenac lies in the range of 4.2–4.5, and when the solution pH < pK_a_, the diclofenac potassium carried a net positive charge due to the protonation, whereas at pH ≅  pK_a_, the deprotonation of the drug started to cause its surface to become negatively charged. Similarly, when pH > pK_a_, the like charges on the catalyst surface and drug molecule started accumulating, causing repulsion between the two, thus decreasing the photodegradation efficiency [71]. 

#### 2.10.2. Effect of Catalyst Dosage

The effect of catalyst dosage on photocatalysis was studied at constant pH by varying the catalyst dosage in the range of 15 mg–35 mg. From Figure 12B, it was observed that the photodegradation efficiency increased with increasing catalytic dosage up to 25 mg, then decreased upon increasing the amount of catalyst above 25 mg. The increased photocatalytic efficiency caused by increasing catalyst dosage can be explained by increased active sites for the adsorption of pollutants, which ultimately produces a more significant number of hydroxyl radicals/superoxide anions. However, when the catalyst dosage was further increased beyond the optimal dosage, it caused a turbidity of the solution, leading to the competition of catalyst molecules to absorb light photons. This condition could have probably caused less penetration and more light scattering, thus decreasing the catalytic efficiency [72].

#### 2.10.3. Effect of Initial Drug Concentration

For an efficient photocatalytic process, it is necessary to investigate the optimal initial concentration of pollutants. In this regard, the initial concentration of diclofenac varied from 5 mg/L to 25 mg/L while maintaining the optimum catalyst dosage and pH of the solution. The maximum photodegradation efficiency of 85.84% was achieved at 5 mg/L diclofenac concentration in 180 minutes of light irradiation due to the presence of a rational number of electron–hole pairs. Further increase in initial drug concentration decreased the photodegradation efficiency, as shown in Figure 12C. For instance, the observed photocatalytic efficiencies were 78.6%, 73.6%, 70.4%, and 67.1% using 10 mg/L, 15 mg/L, 20 mg/L, and 25 mg/L concentrations of diclofenac solution, respectively. At higher drug concentrations, the available catalytic sites become occupied with pollutants, and the number of charge carriers produced by the fixed amount of catalyst are also insufficient to interact with such a significant number of drug molecules; as a consequence, we observe a reduced degradation efficiency [73]. 

### 2.11. Reusability

**Figure 13:**
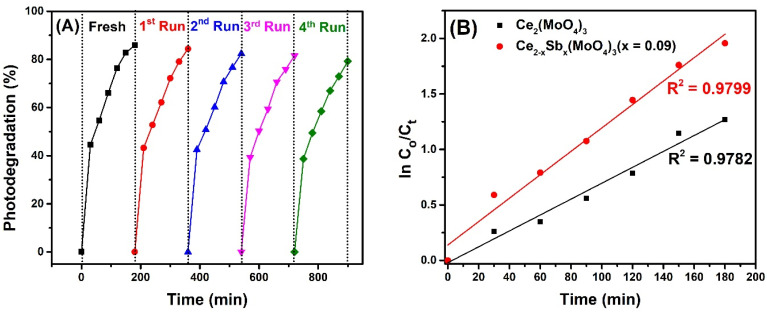
(**A**) Reusability and (**B**) reaction kinetics of photocatalytic degradation of diclofenac potassium using Ce_2−x_Sb_x_(MoO_4_)_3_ (x = 0.09) photocatalyst.

The stability of a photocatalyst in terms of withstanding repeated photocatalytic conditions is one of the desired features in heterogeneous catalysis. Thus, recycling experiments carried out under similar reaction conditions evaluated the reusability of Ce_2−x_Sb_x_(MoO_4_)_3_ (x = 0.09). The fresh catalyst showed an 85.8% photodegradation efficiency under optimized conditions. When the same catalyst was reused for another catalytic run (second cycle), the observed photocatalytic efficiency was 84.31%. Similarly, further reuse resulted in 82.4%, 81.5%, and 79.2% degradation efficiencies for the third, fourth, and fifth cycles, respectively (Figure 13A). These findings present only a 6.6% loss in photocatalytic efficiency after four cycles, suggesting that this material is a sustainable catalyst for water remediation. 

### 2.12. Reaction Kinetics

The kinetics of the photocatalytic reaction was determined by applying a pseudo-first-order kinetic model as depicted in Equation (12):

ln(C_0_/C_t_) = kt (12)

where C_o_ shows the initial concentration of diclofenac, C_t_ represents the concentration of diclofenac after time t, and k denotes the apparent rate constant of the reaction. The plot of ln(C_o_/C_t_) vs. irradiation time (t), which provided a straight line with an R^2^ value approaching to 1, indicated the excellent fit of kinetic data over the pseudo-first-order kinetic model, suggesting that the rate of photocatalytic reaction is proportional to the fraction of the catalyst’s surface interacting with the drug molecule [74]. Moreover, the slope of the plot provided rate constant values of 0.00716 min^−1^ and 0.0105 min^−1^ for Ce_2_(MoO_4_)_3_ and Ce_2−x_Sb_x_(MoO_4_)_3_ (x = 0.09), respectively (Figure 13B). The increased rate constant of Ce_2−x_Sb_x_(MoO_4_)_3_ (x = 0.09) by 2.09 times than Ce_2_(MoO_4_)_3_ is due to the improved textural and surface properties of the former photocatalyst as the rate of reaction improves with more exposed catalytic sites.

### 2.13. Radical Scavenging Study and Photodegradation Mechanism

**Figure 14:**
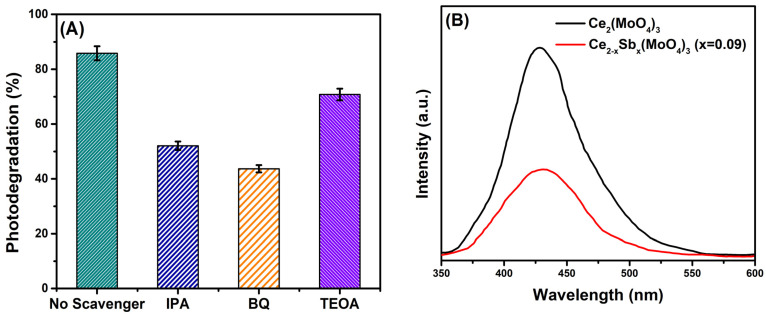

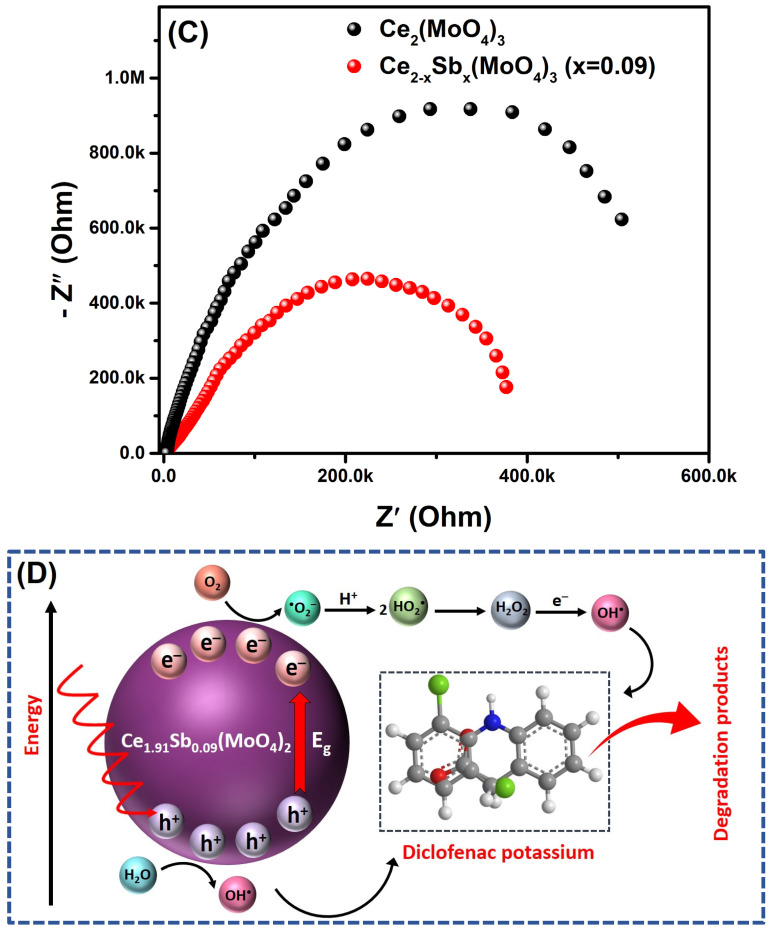
(**A**) Effect of radical scavenging activity, (**B**) PL spectra, (**C**) Nyquist plot, and (**D**) plausible photodegradation mechanism of diclofenac potassium using Ce_2−x_Sb_x_(MoO_4_)_3_ (x = 0.09) photocatalyst.

There are numerous reactive oxidation species, e.g., superoxide anions (•$O_{2}^{-}$), holes (h^+^), and hydroxyl radicals (OH•), which are produced during photocatalytic reaction and bring about the degradation of pollutants. To investigate the concerned reactive species involved in the photodegradation process, the radical scavenging studies were performed using benzoquinone (BQ), isopropyl alcohol (IPA), and triethanolamine (TEOA) for scavenging superoxide anion, hydroxyl radical, and holes, respectively. The photocatalytic reaction without any scavenger was used as control, and maximum degradation efficiency (85.84%) was achieved owing to the contribution of all reactive species produced. When TEOA was used as a scavenger, only a slight decrease in photodegradation efficiency (70.9%) was observed, revealing that holes played a minimal role in the degradation process. However, 43.7% and 52.2% photodegradation efficiencies were recorded when the photocatalytic reaction was carried out in the presence of BQ and IPA, respectively (Figure 14A). This outcome demonstrated that BQ strongly hampered the catalytic activity as it confined the produced superoxide radicals, which were supposed to bring about photocatalytic degradation. Similarly, IPA also caused a significant decrease in degradation efficiency as hydroxyl radicals were scavenged in this experiment. It indicated that superoxide radicals were the dominant active species involved in the photodegradation process followed by hydroxyl radicals, consistent with the findings of Jiménez-Salcedo et al. [75]. 

The recombination of photo-induced charge carriers was studied via photoluminescence (PL) spectroscopy. The PL spectrum of Ce_2_(MoO_4_)_3_ showed an intense emission peak, indicating the fast recombination rate of charge carriers; contrarily, the intensity of the emission peak declined to a greater extent for Ce_2−x_Sb_x_(MoO_4_)_3_ (x = 0.09) (Figure 14B). It demonstrated that Sb doping has efficiently suppressed the recombination of charge carriers as fluorescence intensity is directly related to the electron–hole recombination rate, which could improve photocatalytic activity [76]. The slow recombination, and thus, the significant separation between charge carriers were further supported by the Nyquist plot obtained from impedance data (Figure 14C). This plot implies that the smaller the radius of the semicircle, the lower the charge transfer resistance and the greater the separation efficiency of charge carriers at the catalyst’s interface [77]. The smaller semicircle of the Ce_2−x_Sb_x_(MoO_4_)_3_ (x = 0.09) Nyquist plot than the semicircle of Ce_2_(MoO_4_)_3_ proved the improved photocatalytic performance of Ce_2−x_Sb_x_(MoO_4_)_3_ (x = 0.09) over Ce_2_(MoO_4_)_3_ in terms of good charge transfer, while maintaining suitable charge separation.

Figure 14D illustrates the probable mechanism of our photocatalytic reaction. The process initiates when a photocatalyst (Sb-doped cerium molybdate) is irradiated with light of sufficient energy. It causes the migration of electrons from the valence band of the catalyst to the conduction band; thus, holes are produced in the valence band (VB), and electrons are found in the conduction band (CB) (Equation (13)). The generated e^−^/h^+^ pairs are primary active species that migrate to the catalyst’s surface, producing reactive species through redox reactions. For instance, the electrons react with molecular oxygen and give rise to superoxide anion (•$O_{2}^{-}$), whereas holes (h^+^) attack the water molecule and convert it into hydroxyl radical (OH•) and H^+^ according to Equations (14) and (15). The H^+^ reacts with formerly produced •$O_{2}^{-}$ to produce •HO_2_, which eventually converts into H_2_O_2_, followed by its splitting into OH• (Equations (16)–(18)). These OH• radicals, along with •$O_{2}^{-}$, are known as dominant species responsible for the degradation of pollutant molecules into relatively simple and less harmful compounds, as shown through Equations (19) and (20) [78]:

(13) $${Ce}_{1.91}{Sb}_{0.09}(MoO_{4}{)}_{3}+hv\to h_{VB}^{+}+e_{CB}^{-}$$

(14) $$e_{CB}^{-}+O_{2}\to {•O}_{2}^{-}$$

(15) $$h_{VB}^{+}+H_{2}O\to OH^{•}+H^{+}$$

(16) $${•O}_{2}^{-}+H^{+}\to {HO}_{2}^{•}$$

(17) $$2{HO}_{2}^{•}\to H_{2}O_{2}+O_{2}$$

(18) $$e_{CB}^{-}+H_{2}O_{2}\to {2OH}^{•}$$

(19) $${OH}^{•}+Diclofenac\;potassium\to Degradation\;products$$

(20) $${•O}_{2}^{-}+Diclofenac\;potassium\to Degradation\;products$$

## 3. Materials and Methods

### 3.1. Materials

The chemicals used were cerium (III) nitrate hexahydrate (Ce (NO_3_)_3_∙6H_2_O, 99.0%), sodium molybdate dihydrate (Na_2_MoO_4_∙2H_2_O, 99.0%), antimony (III) nitrate (Sb(NO_3_)_3_, 97.0%), and sodium hydroxide (NaOH, 99%). All chemicals were of analytical grade and obtained from Sigma Aldrich (St. Louis, MO, USA). The raw form of diclofenac potassium was provided by Pharmagen Limited (Lahore, Pakistan). Deionized water was used in all experiments for the dissolution of precursors. 

### 3.2. Synthesis of Cerium Molybdate and Sb-Doped Cerium Molybdate

Ce_2−x_Sb_x_(MoO_4_)_2_ (x = 0.00, 0.01, 0.03, 0.05, 0.07, 0.09) was synthesized via a co-precipitation method as reported by Mayuranathan et al. [79]. Well-prepared solutions of cerium nitrate hexahydrate (0.2 M) and sodium molybdate dihydrate (0.3 M) were homogenously mixed together and stirred for 45 min. Subsequently, sodium hydroxide (4 M) was added drop by drop in the above solution until pH became 9. At this stage, yellow-colored precipitates of cerium molybdate appeared in the reaction mixture. The precipitates were filtered, washed, and oven dried at 98 °C for 4 h. The final product was ground to fine powder and subjected to calcination at 650 °C for 4–5 h. For doped compositions, the stoichiometric amount of antimony nitrate was added in the homogenous solution of cerium nitrate and sodium molybdate followed by the similar steps as described for undoped cerium molybdate. The detailed procedure for the synthesis of antimony-doped cerium molybdate is illustrated in Figure 1.

### 3.3. Characterization

The optical properties of synthesized materials were studied via ultraviolet–visible spectroscopy (PerkinElmer spectrophotometer λ–35, Waltham, MA, USA). Fourier transform infrared spectroscopy (Cary 630–Agilent technologies) and Raman spectroscopy were employed to investigate the functional groups and bond formation in fabricated materials. The chemical states of synthesized materials were examined via X-ray photoelectron (XPS) spectroscopy. For structural study, X-ray crystallography (D2 Phaser X-ray diffractometer, Bruker, Cu Kα radiation (*λ* = 1.5406 Å)) technique was used, whereas surface properties were studied using Brunauer–Emmett–Teller (BET) analysis (Micromeritics (Gemini VII, 2390, Norcross, GA, USA)). The samples were degassed at 300 °C for 3 h in an N_2_ environment prior to BET analysis. For dielectric studies, powdered samples were first converted into uniform pallets using hydraulic press (operating pressure = 160 MPa; pressing time = 10 min) and then obtained pallets were analyzed at room temperature using impedance analyzer (6520B, Make: Wayne Kerr, Bognor Regis, West Sussex, UK). 

### 3.4. Photocatalytic Activity

The photocatalytic activity of Ce_2−x_Sb_x_(MoO_4_)_2_ (0.00 ≤ x ≤ 0.09) was investigated against potent anti-inflammatory drug diclofenac potassium using 8 W UV lamp as light irradiation source. Each of the synthesized material (20 mg) was added as photocatalyst into the aqueous solution of diclofenac potassium (5 mg L^−1^, 50 mL) in separate photocatalytic runs. The working solution was equilibrated by stirring it continuously for half an hour in the dark, prior to the light exposure. Afterward, the suspension was irradiated with light under constant stirring and uniform conditions. The analytical samples were taken out from the reaction mixture after equal intervals of 30 min, followed by centrifugation to separate the photocatalyst from drug solution. The supernatant was analyzed through UV–Vis spectrophotometer and concentration of residual drug was measured. The obtained data were utilized to calculate the photodegradation efficiency (%) using the following equation (Equation (21)) [80]:

(21) $$\mathrm{Photodegradation}\;(\%)=[(C_{o}-C_{t})/C_{t}]\times 100$$

where C_t_ is the concentration of drug solution at certain time interval of light irradiation, whereas C_o_ is the equilibrium concentration of drug solution obtained before light exposure. 

#### 3.4.1. Optimization of Photocatalytic Operating Parameters

Catalyst dosage, initial concentration of pollutant, and pH of the reaction mixture are some of the crucial factors which affect the efficiency of photocatalytic process; therefore, it is essential to study these parameters to obtain maximum photodegradation efficiency. Amid various synthesized compositions, the material having maximum photocatalytic efficiency was selected as the preferred catalyst to perform further photocatalytic studies. The influence of pH was examined by changing the solution pH from 2 to 11 while keeping other parameters constant, i.e., initial drug concentration = 5 mg/L and catalyst loading = 20 mg. HCl (1 M) and NaOH (1 M) were used to maintain pH. The effect of catalyst dosage was investigated by varying the catalyst amount from 15 mg to 35 mg at pH = 4 and an initial drug concentration of 5 mg/L. In the following series of experiments, initial drug concentration was varied from 5 mg/L to 25 mg/L at optimum catalyst dosage of 5 mg and pH = 4. 

#### 3.4.2. Reusability

For reusability test, the used photocatalyst was recovered from the drug solution via centrifugation after the first photocatalytic experiment. The photocatalyst was washed with deionized water, followed by ethanol, and then oven-dried at 60 °C for 24 h. The dried photocatalyst was used for the second photocatalytic run under the same experimental conditions and the same procedure was repeated until a total of five photocatalytic cycles were completed. 

#### 3.4.3. Radical Scavenging Experiment

The dominant active species involved in the photodegradation of diclofenac drug was identified via radical scavenging experiments. Each scavenger (5 mL) was separately mixed with the drug solution, and the whole catalytic process was repeated under identical conditions. The scavengers used were isopropyl alcohol (IPA, 0.01 M), triethanolamine (TEOA, 0.01 M) and benzoquinone (BQ, 0.01 M) to quench hydroxyl radicals (OH^•^), holes (h^+^), and superoxide anions (^•^O_2_^−2^), respectively. 

## 4. Conclusions

Sb-doped cerium molybdate (Ce_2−x_Sb_x_(MoO_4_)_3_) with variable concentrations of antimony (x = 0.00, 0.01, 0.03, 0.05, 0.07, 0.09) were successfully synthesized via facile co-precipitation method and characterized using various analytical techniques. All materials showed a well-crystallized monoclinic phase of cerium molybdate with reduced crystallite sizes upon Sb doping. The optical band gaps were also narrowed down, and materials became more porous by increasing Sb content in the cerium molybdate host lattice. These modifications remarkably improved the dielectric properties of the materials; in particular, the Ce_2−x_Sb_x_(MoO_4_)_3_ composition with x = 0.09 showed the highest dielectric constant (2.856 × 10^8^) among all synthesized materials with small current leakage (1.647) and excellent AC conductivity (17.141 S m^−1^). In addition, the photocatalytic efficiency of synthesized materials was also appreciably enhanced by increasing Sb content. The Ce_2−x_Sb_x_(MoO_4_)_3_ (x = 0.09) showed a maximum degradation efficiency of 85.8% against diclofenac potassium under optimized conditions and followed pseudo-first-order kinetics. These findings suggest the potential use of the proposed materials in charge storage applications and environmental remediation to address challenges of utmost importance in the present and upcoming era. 

## Figures and Tables

**Table 1:** Calculated XRD parameters for Ce_2−x_Sb_x_(MoO_4_)_3_ using variable dopant concentrations.

Composition	D (nm)	δ (lines/m^2^)	ε	Crystallinity (%)
x = 0.00	40.29	6.16 × 10^−13^	0.000467	70.5
x = 0.01	36.86	7.36 × 10^−13^	0.000471	77.6
x = 0.03	34.98	8.17 × 10^−13^	0.000502	85.2
x = 0.05	32.63	9.39 × 10^−13^	0.000586	83.2
x = 0.07	31.27	1.02 × 10^−12^	0.000559	70.7
x = 0.09	29.09	1.18 × 10^−12^	0.000646	85.4

**Table 3:** Comparison of dielectric constant (ε′), loss tangent (tanδ), and AC conductivity (σ_ac_) of synthesized composition Ce_2−x_Sb_x_(MoO_4_)_3_ (x = 0.09) with previously reported related materials.

Material	Frequency	Dielectric Constant	Dielectric Loss	AC Conductivity	Reference
BaCe_2_(MoO_4_)_4_	7.44 GHz	12.3	–	–	[55]
Ce_2_(MoO_4_)_2_(Mo_2_O_7_)	12.73 GHz	10. 69	1.88 × 10^−4^	–	[7]
La_6−x_Sm_x_MoO_12_ (x = 0.8)	2 MHz	32.40	–	1.5 × 10^−5^ S cm^−1^	[56]
Ce_2_[Zr_1−x_(Al_1/2_Nb_1/2_)x]_3_(MoO_4_)_9_ (x = 0.04)	12.97 GHz	10.54	1.04 × 10^−4^	–	[57]
Ce_2_[Zr_1−x_(Cr_1/2_Nb_1/2_)_x_]_3_(MoO_4_)_9_ (x = 0.06)	13.069 GHz	10.56	1.383 × 10^−3^	–	[58]
Ce_2_[Zr_0_._98_(Cr_0_._5_Sb_0_._5_)_0_._02_]_3_(MoO_4_)_9_	13.18 GHz	10.53	1.42 × 10^−3^	–	[59]
Ce_2−x_Sb_x_(MoO_4_)_3_ (x = 0.09)	20 MHz	8.441 × 10^4^	0.1826	17.141 S m^−1^	This work

**Table 4:** Comparison of photocatalytic degradation efficiencies of various reported catalysts against diclofenac potassium.

Catalyst	Catalyst Amount	Diclofenac Concentration	Light Source	Irradiation Time	Photocatalytic Efficiency	Rate Constant	Ref.
N, S co-doped TiO_2_@MoS_2_	1 g/L	5 mg/L	LED lamp (60 W)	150 min	64%	0.002 min^−1^	[65]
Bi_2_MoO_6_ nanofilms	–	10 ppm	Sunlight	60 min	98.57%	0.052 min^−1^	[66]
MoS_2_/Cd_0_._9_Zn_0_._1_S	25 mg	20 μM	Xenon lamp	30 min	86%	–	[67]
V_2_O_5_/B–co-doped g–C_3_N_4_	2 g/L	5 mg/L or 10 mg/L	Xenon lamp (150 W)	100 min	100%	–	[68]
Ce–B–TiO_2_	–	5 ppm	Mercury lamp (125 W)	180 min	99%	0.388 min^−1^	[69]
Ce_2−x_Sb_x_(MoO_4_)_3_ (x = 0.09)	25 mg	5 mg/L	UV lamp (8 W)	180 min	85.8%	0.0105 min^−1^	This work

## Data Availability

Data are contained within the article.
